# Supplementary material for: Structural variability of multifunctional proteins indicates frequent stochastic evolution of protein oligomers
Source: Commun Biol. 2025 Dec 13;8:1820. doi: 10.1038/s42003-025-09183-5 (PMC12749353; doi:10.1038/s42003-025-09183-5)
Supplement: Supplementary file 1 — Supplementary Information [file 42003_2025_9183_MOESM1_ESM.pdf]

Supplementary Figures to “Structural variability of multifunctional proteins indicates frequent stochastic evolution of protein oligomers”

by György Abrusán *[1] and Aleksej Zelezniak [1,2,3]

[1] Randall Centre for Cell and Molecular Biophysics, School of Basic and Medical Biosciences, King’s College London, New Hunt’s House, Great Maze Pond, London, SE1 9RT, UK

[2] Department of Life Sciences, Chalmers University of Technology, Kemivägen 10, SE41296, Gothenburg, Sweden

[3] Institute of Biotechnology, Life Sciences Centre, Vilnius University, Sauletekio al. 7, LT10257, Vilnius, Lithuania

* corresponding author
email: gyorgy.abrusan@kcl.ac.uk

On all panels, numbers in parentheses are p-values corrected for multiple testing (FDR).

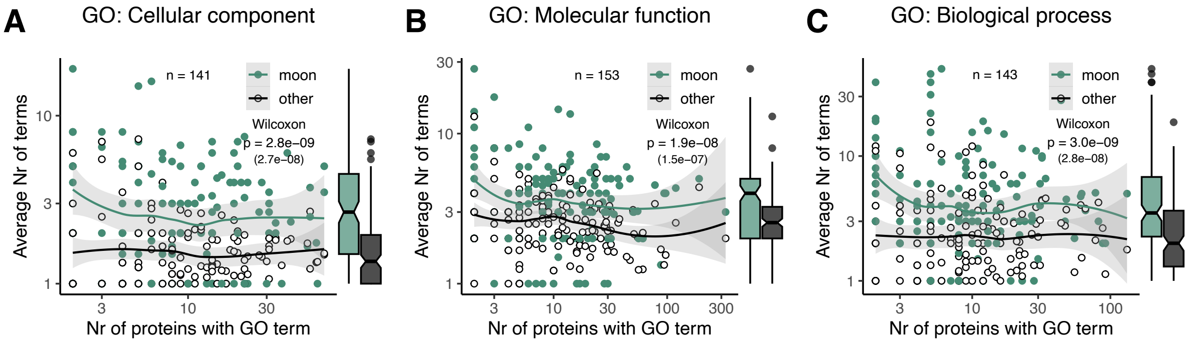

Supplementary Figure 1. GO annotation of proteins in moonlighting orthogroups. Within the same orthogroup, known moonlighting proteins have significantly higher numbers of GO terms than proteins that are absent in the moonlighting dataset, in all three GO categories. Each orthogroup is represented by two points, one with the average of known moonlighting proteins in the orthogroup, and one with the average of proteins currently not known to be moonlighting. The number of terms of the latter proteins is likely to be underestimated though, because many of them are also likely to be moonlighting (see Figure 2.)

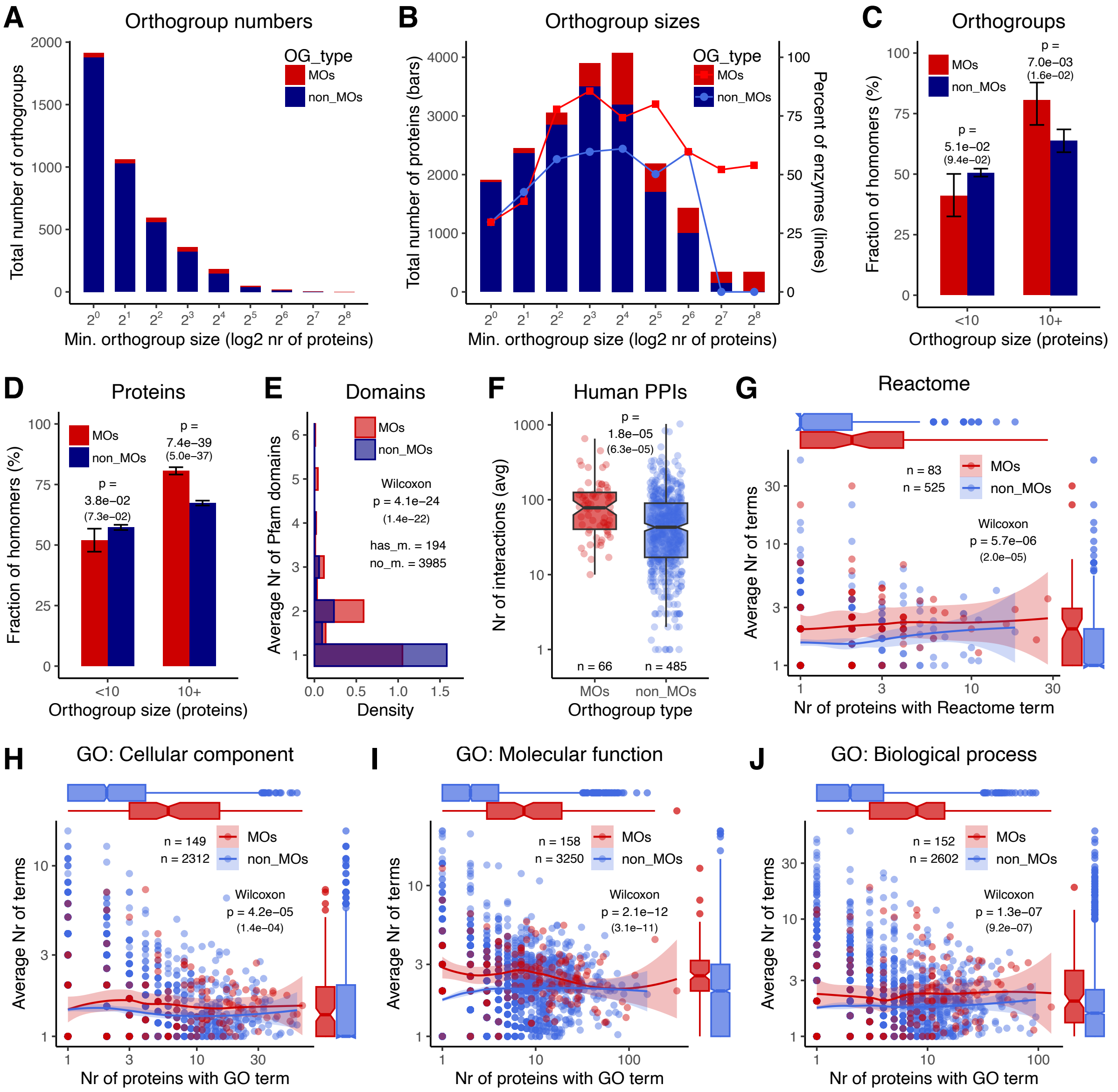

Supplementary Figure 2. The same as Figure 2, but excluding known moonlighting proteins from MOs on all panels. None of the patterns of Figure 2 are changed qualitatively, on average, MOs are annotated with more functions.

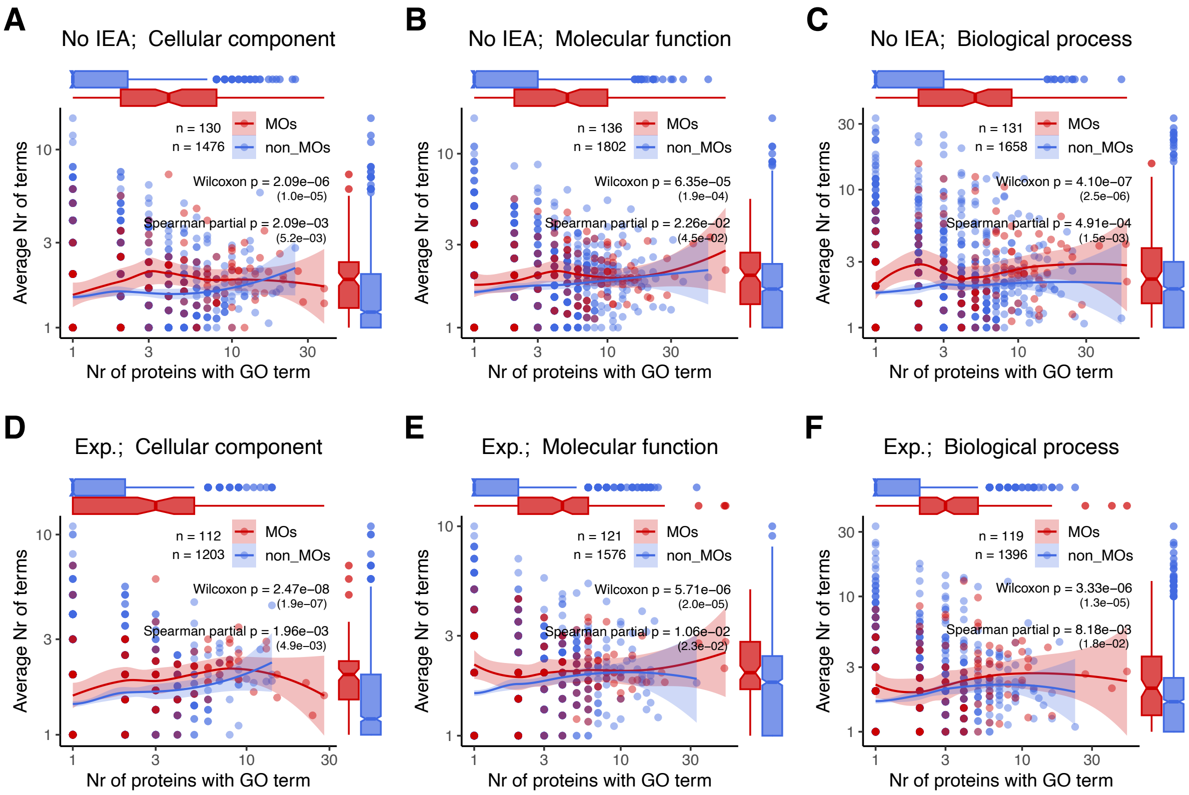

Supplementary Figure 3. The difference between MOs and non-MOs remains significant when only subsets of GO terms are used. **A-C)** Terms excluding the ones with IEA evidence code ("**I**nferred from **E**lectronic **A**nnotation"). **D-E)** Terms with experimental evidence codes only, which included the "EXP", "IDA", "IPI", "IMP", "IGI", "IEP", "HTP", "HDA", "HMP", "HGI", and "HEP" evidence codes. In both cases the difference is smaller than for the full set of terms, nevertheless remains significant.

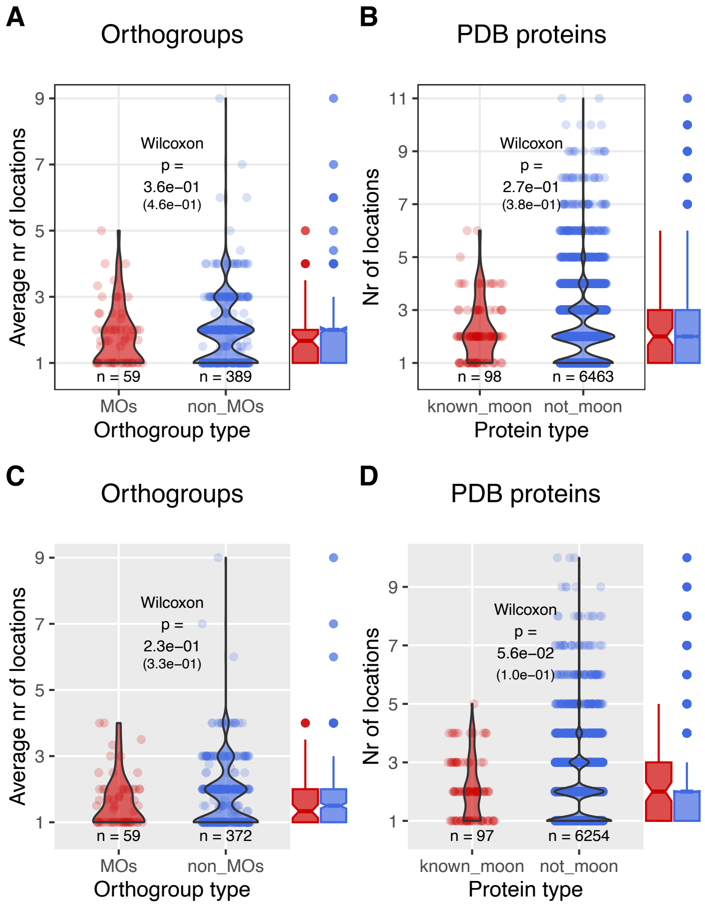

Supplementary Figure 4. The number of subcellular locations of human proteins. **A and B)** All subcellular locations of the Human Protein Atlas (HPA). No clear difference is visible between the MOs and non-MOs (A), or when all human proteins in the PDB are used (B), including the proteins that are not present in the orthogroups. **C and D)** The same as on panels A and B, but excluding the locations annotated as "Uncertain", which, at least partly, are likely to include also protein mislocalizations in the HPA cell lines.

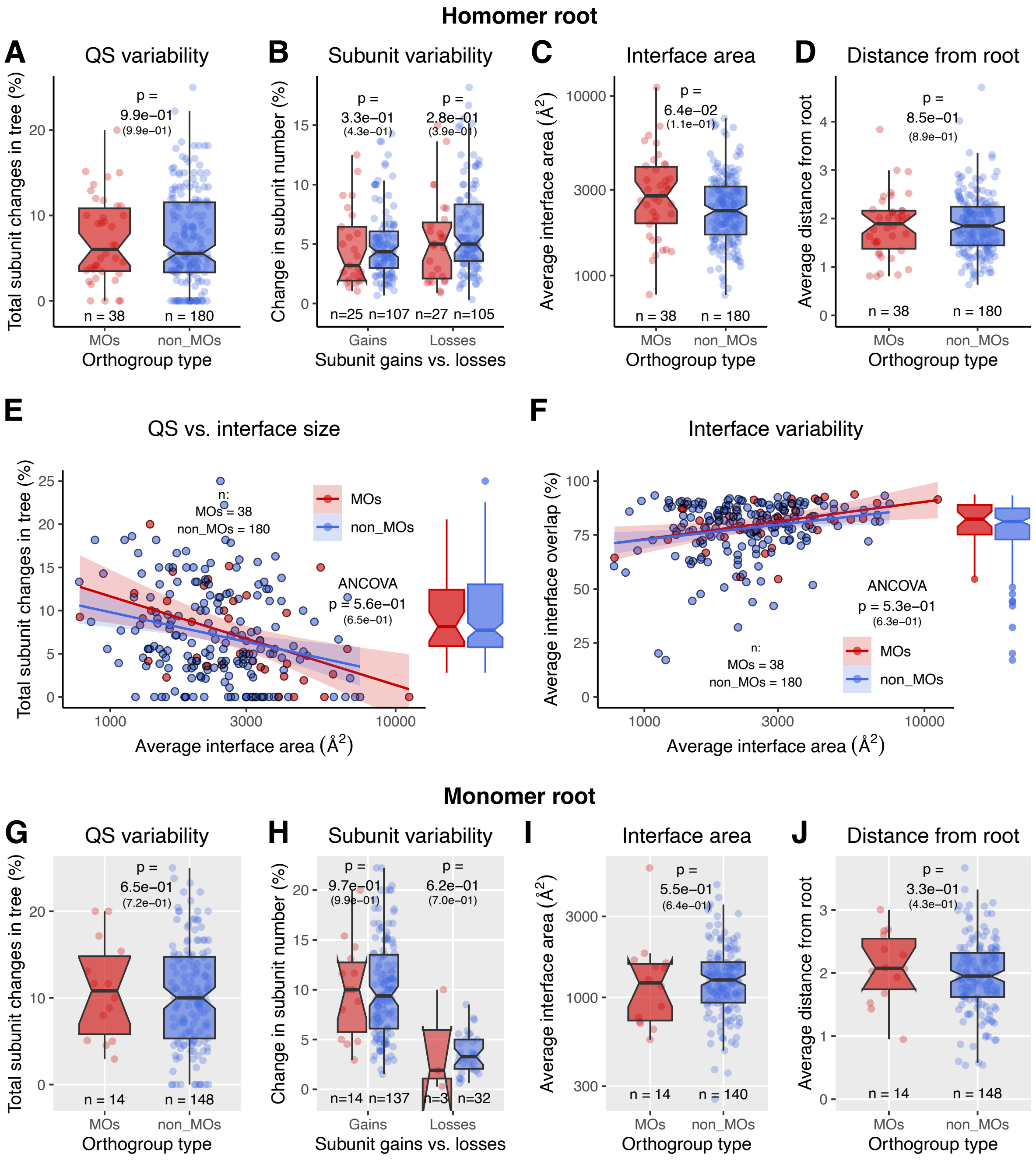

Supplementary Figure 5. The rate of change of quaternary structure in the phylogenetic trees, using maximum parsimony to estimate the ancestral state of the nodes. Similarly Figure 3, no significant differences could be detected in the rate of QS changes, neither in the trees with homomer root (A-F), nor in the ones with monomer root (G-J).

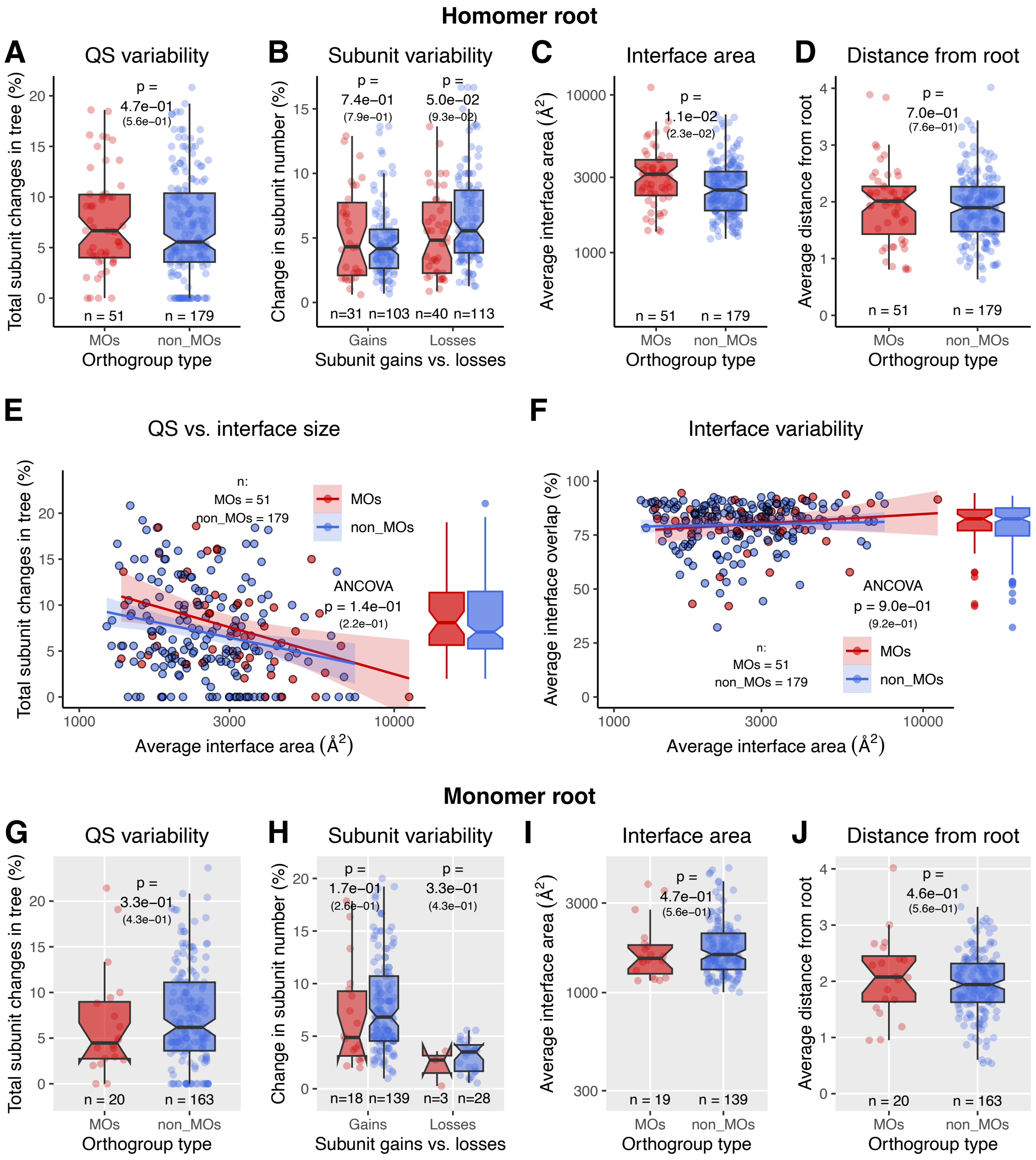

Supplementary Figure 6. The rate of change in quaternary structure in the dataset where homomers with small (<1000 Å^2) interfaces were included among the monomers. No significant differences could be detected in the rate of QS changes, neither in the trees with homomer root (A-F), nor in the ones with monomer root (G-J).

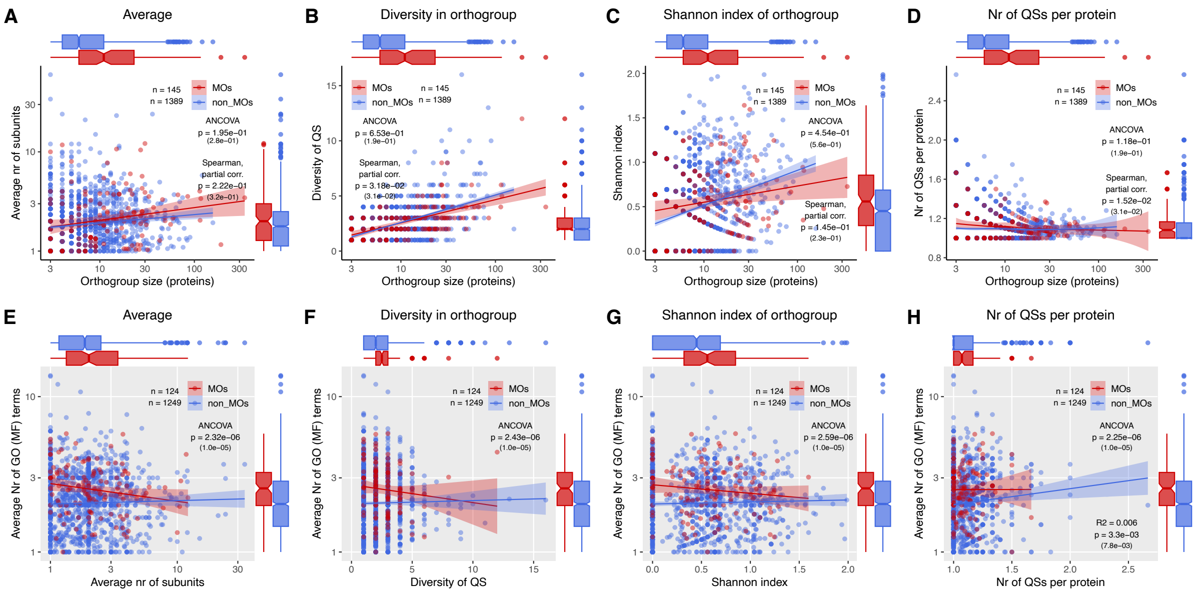

Supplementary Figure 7. Quaternary structure diversity metrics vs. GO term diversity in the dataset where homomers with small (<1000 Å^2) interfaces were included among the monomers. The patterns are largely the same as in the original dataset, MOs and non-MOs show no clear difference in QS variability (panels A-D), and the differences in GO terms are largely unexplained by QS variability metrics (panels E-H).

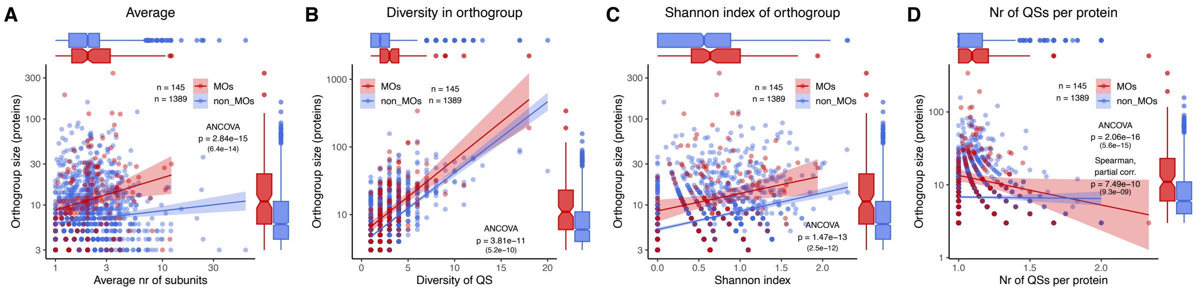

Supplementary Figure 8. The same as Figure 4 E-H, but with the x-y axes swapped. For all diversity metrics, at the same level of diversity, MOs have consistently more proteins.

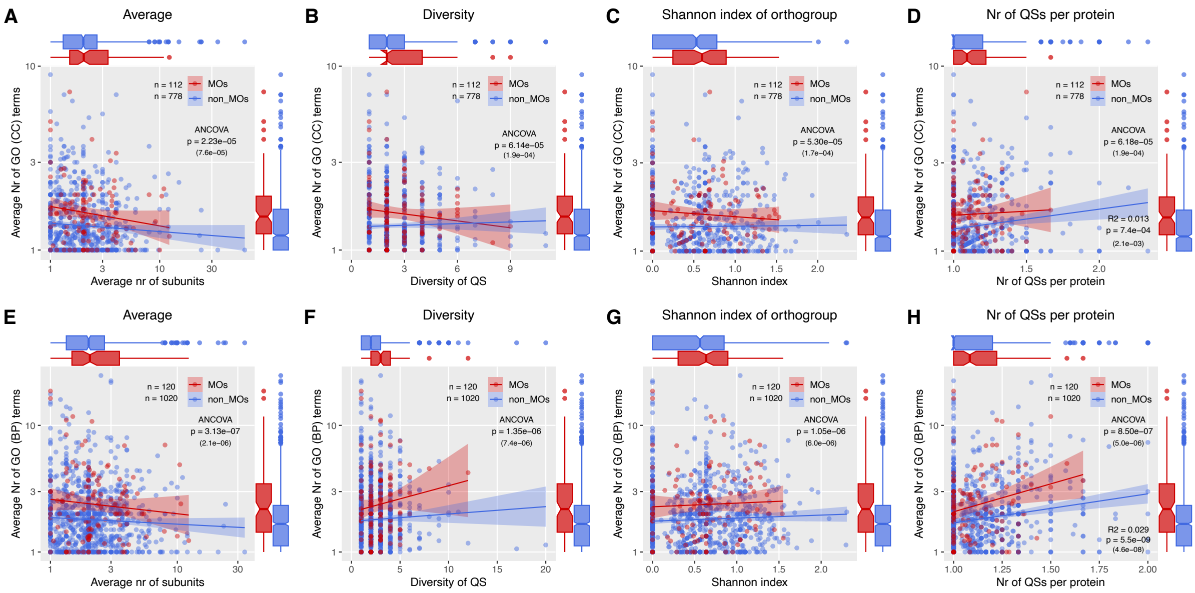

Supplementary Figure 9. Correlations between QS diversity metrics and Cellular Component and Biological Process GO terms. Similarly to the Molecular Function terms, QS diversity metrics do not explain the difference between MOs and non-MOs, and a positive correlation could be detected only for the Nr of QSs per protein (panels D and H), which explain 1-2% of variance.

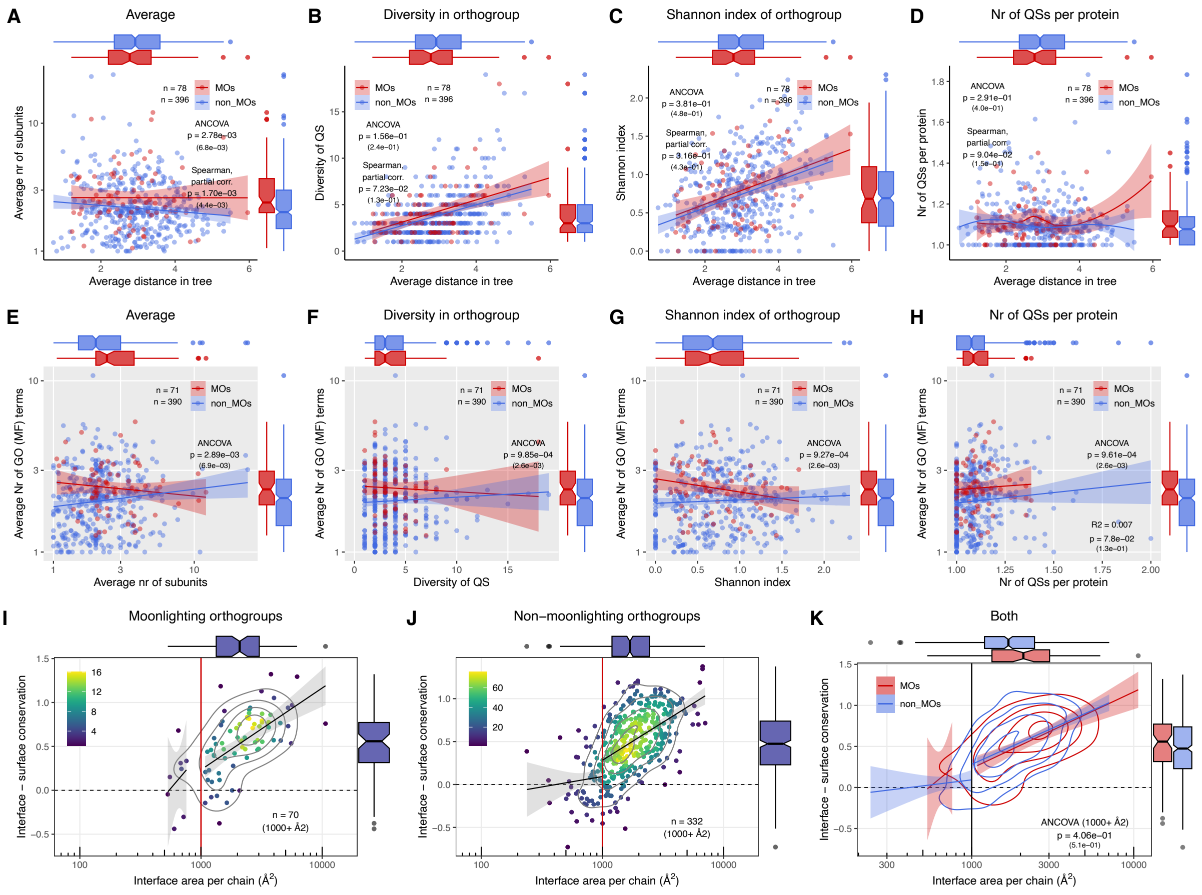

Supplementary Figure 10. Quaternary structure diversity and the average distance of proteins within the phylogenetic trees scales similarly in MOs and non-MOs. **A-D)** Diversity, Shannon index and the average number of quaternary structures per protein is not significantly different between the two groups, the exception being the number of subunits (A), which, similarly to Figure 2E and 2F, is significantly higher in MOs. **E-H)** The diversity indices do not explain the difference in GO Molecular Function terms. **I-K)** Interface conservation is not different between the two groups.

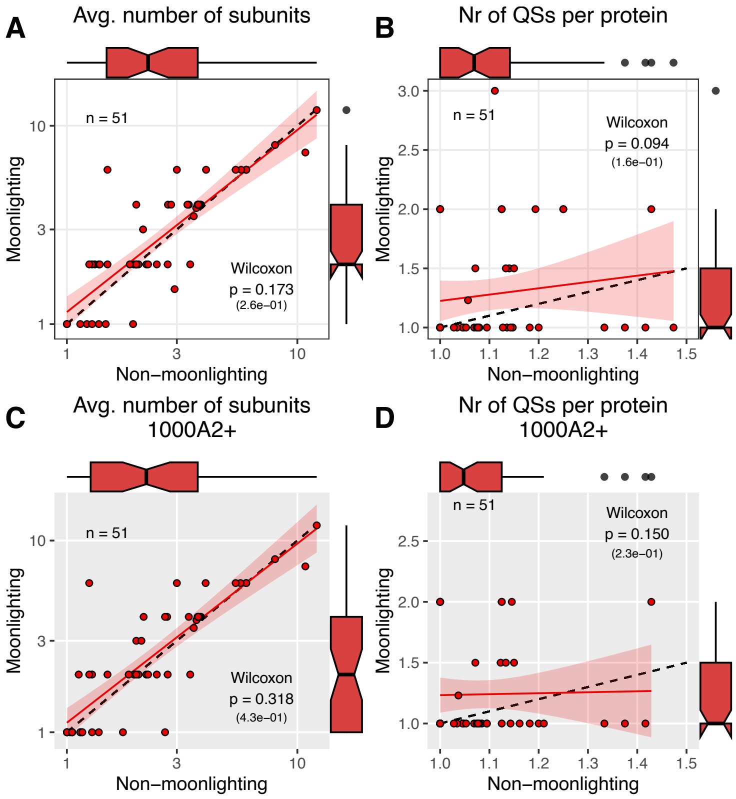

Supplementary Figure 11. Comparison of known moonlighting proteins and proteins currently not annotated as moonlighting within the same orthogroups (MOs). Neither the average number of subunits (panel A), nor the Nr of QSs per proteins are different (panel B), irrespectively whether the unaltered set of homomers (panels A-B), or the modified set, where homomers with interfaces <1000 Å^2 were included among monomers is used (panels C-D)

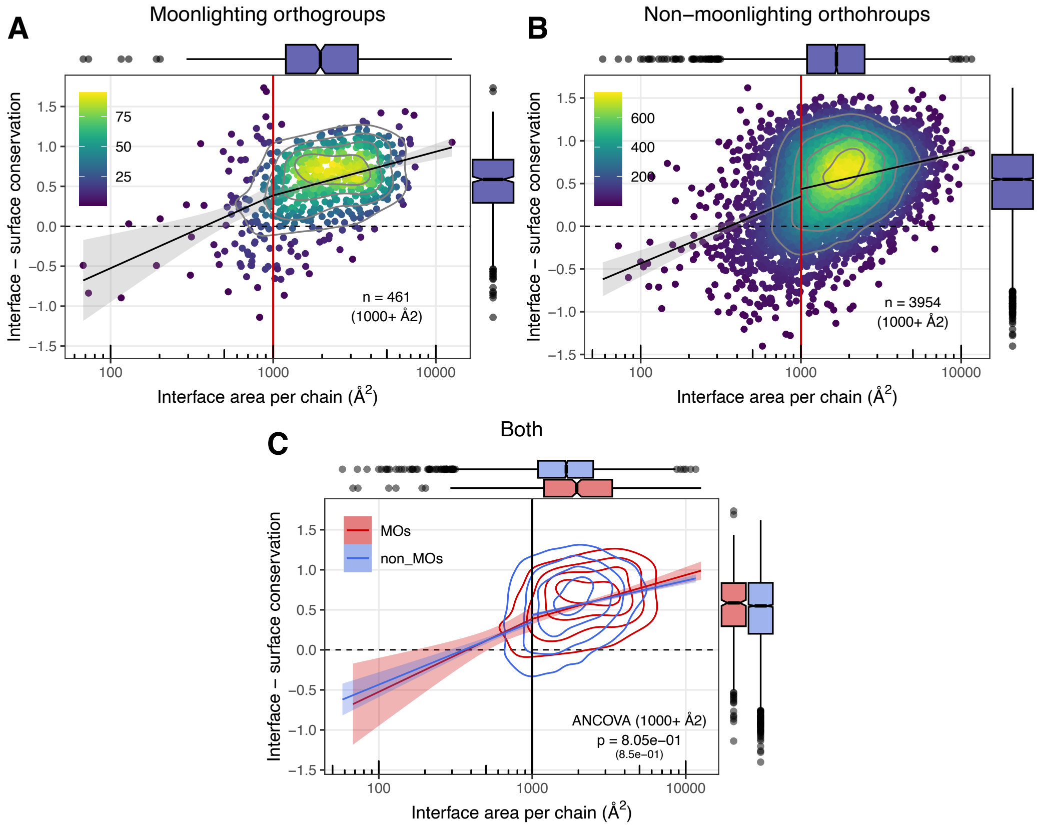

Supplementary Figure 12. Conservation of interfaces, using clusters with min 30% sequence similarity. Each point represents the centroid of a cluster. No significant difference could be detected between the elements of MOs and non-MOs (panel C).
