# Supplementary material for: Structural variability of multifunctional proteins indicates frequent stochastic evolution of protein oligomers
Source: Commun Biol. 2025 Dec 13;8:1820. doi: 10.1038/s42003-025-09183-5 (PMC12749353; doi:10.1038/s42003-025-09183-5)
Supplement: Supplementary file 2 — Description of Additional Supplementary Files [file 42003_2025_9183_MOESM2_ESM.docx]

Description of Additional Supplementary Files

**File name:** SuppFigures.pdf

**Description:** Supplementary Figures 1-12.

**File name:** SuppData.xlsx

**Description:** Supplementary data (tables) 1-3.

Data S1. The list of proteins in the orthogroups.

Data S2. The list of moonlighting proteins in the orthogroups.

Data S3. The list of moonlighting proteins in the orthogroups, excluding predicted proteins from MoonDB.

|  |  |
| --- | --- |
